# Accurate prediction of UV absorption in zirconia–alumina mixed oxide (ZrAl_4_O_8_) via GW–BSE calculations

**DOI:** 10.1007/s00894-026-06939-6

**Published:** 2026-09-09

**Authors:** Alexandre B. Rocha, Angela S. Rocha

**Affiliations:** 1 Chemistry Institute, Federal University of Rio de Janeiro, Avenida Athos da Silveira Ramos, 149, Centro de Tecnologia, Rio de Janeiro, Rio de Janeiro 21941-909 Brazil; 2 Chemistry Institute, Rio de Janeiro State University, Rua São Francisco Xavier, 524, Maracanã, Rio de Janeiro, Rio de Janeiro 20550-900 Brazil

**Keywords:** Acid–base catalyst, DFT, Spectroscopy

## Abstract

**Context:**

Zirconia is a technologically relevant material widely employed in catalysis owing to its thermal stability, acid–base surface properties, and optical response, enabling applications both as a solid acid catalyst and as a photocatalytic material. Even so, the relatively low specific surface area of bulk zirconia may limit its catalytic performance, making supported systems and mixed oxides attractive alternatives for enhancing dispersion and surface accessibility. Among these, zirconia–alumina mixed oxides have attracted significant attention because of the strong interfacial interactions established between both phases, which may involve a migration of Zr^4^⁺ species into the subsurface region of alumina, leading to the formation of structurally stable mixed oxide domains. This behavior has been associated with modifications in the electronic structure and surface reactivity of the material. In the present work, the UV absorption spectrum of the mixed oxide was calculated, and reasonable agreement was achieved with the measured gap and spectrum, reinforcing the existence of the mixed oxide. The bandgap was determined as 6.47 eV, close to the experimental values, and the simulated spectrum shows good agreement with the experimental profile.

**Methods:**

The geometry of the mixed oxide was optimized with and without constraint at the DFT/PBE level with periodic boundary conditions, a plane-wave basis set for valence electrons, and projected augmented waves (PAW) to treat core electrons. Excited-state optical properties were evaluated by solving the Bethe–Salpeter equation (BSE) on top of GW quasiparticle energies, allowing an accurate description of electron–hole interactions and optical transitions. All calculations were done in VASP software.

**Supplementary Information:**

The online version contains supplementary material available at https://doi.org/10.1007/s00894-026-06939-6.

## Introduction

Zirconia (ZrO_2_) has interesting catalytic properties, since it can be used as an acidic catalyst [1–3] and as a semiconductor in photocatalyst processes as nanoparticles [4, 5]. Nonetheless, its high cost, difficult phase control and unfavorable textural properties, such as low pore volume and surface area, limit its use as catalysts in pure form. A possible way to circumvent these features is to support it in materials as *γ*-alumina (Al_2_O_3_). The resulting ZrO_2_/Al_2_O_3_ has been used mainly as supports or acid–base catalysts for processes such as hydrolysis, reforming, hydrogenation [6–8].

A relevant issue that arises in these zirconia-based materials supported on alumina is the formation of mixed oxides by the migration of Zr^4+^ ions through the alumina lattice. Faro et al. [9] prepared alumina-supported zirconia by impregnation with benzene solutions of zirconium acetylacetonate to study the surface properties of the materials, mainly acid sites. The characterization of the samples has shown, though, that after calcination at 823 K, there was no ZrO_2_ phase formed even for loadings corresponding to more than 80% of the theoretical monolayer. They suggested that there was a mixing between zirconia and alumina, by migration of zirconium ions to the subsurface layers of the support. In another paper [10], a theoretical–experimental study was conducted in order to corroborate the zirconium migration hypothesis. DFT calculation was performed, and a structure for a mixed oxide was proposed. This structure was used to simulate an EXAFS profile, which led to a very good agreement with the measured spectrum.

The evidence of Zr and Al mixed oxides was also suggested in other papers [11–14], but ZrO_2_ and Al_2_O_3_ phases were also identified, depending on the synthesis methodology used. For instance, despite the strong interaction between Zr and Al oxides, Korhonen et al. reported that no mixed oxide formation occurs [15].

Interestingly, in their first paper, Faro et al. [9] also characterized different samples of mixed zirconia–alumina phases by diffuse reflectance spectroscopy (DRS), among other techniques. The results showed that neither the measured bandgap nor the UV-visible spectral profile corresponded to those of pure zirconia. Therefore, these findings provide additional evidence for the formation of a zirconia–alumina mixed phase.

The most ubiquitous method used to assess structural properties for many solid materials is, in the present day, indisputably the density functional theory (DFT). Nonetheless, this approach fails to determine bandgaps. This can be understood by the fact that there exists no formal justification to interpret the DFT eigenvalues as quasi-particle energies. The alternative available is the GW approximation [16, 17]. The most important limitation to the use of this method in the last decades is its impressive computational cost. By the time the ZrAl_4_O_8_ structure was proposed [10], it was very challenging, perhaps impossible, to perform GW calculations, since the use of the simplifying plasmon-pole method, common in early implementations, is not recommended for structures containing transition metals [17], although the generalized version due to Hybertsen and Louie (HL–GPP) [18] is more appropriate for inhomogeneous systems like the ones containing transition metal atoms, leading to much better agreement with experimental values as, for instance, in MoS_2_ [19].

Now comes the time these methods (GW and GW–BSE) have, due to advances in algorithms and hardware, become feasible. The approach used here relies on the VASP implementation, which calculates the dielectric matrix in full frequency.

While GW is derived from the equations of motion of a one-particle Green’s function, the Bethe–Salpeter equation (BSE) is derived from a two-particle Green’s function. The so-called GW–BSE consists in performing BSE calculations with a previous GW calculation furnishing quasi-particle (QP) energies to compute the free electron–hole transition and the screened Coulomb potential. The resulting Bethe–Salpeter equation can be set in the form of a generalized eigenvalue problem, similar to Cassida’s equation in TDDFT. Details can be found in a recent review [20].

In the present work, we have performed GW and GW–BSE calculations in order to calculate the bandgap and obtain the UV–vis spectrum for the model phase zirconia–alumina previously proposed [9]. The model phase, formula ZrAl_4_O_8_, has a defective spinel structure as explained in what follows.

## Computational details

The structure of the ZrAl_4_O_8_ phase was determined before [10] by using density functional theory (DFT) within the local density approximation (LDA) and periodic boundary conditions. In the present work, the structure, designated here as OT following the designation of the previous work [10], was derived from a model structure for gamma-alumina as a defective spinel, described elsewhere [21], by isomorphic substitution of Al^3+^ by Zr^4+^ in certain positions and the consequent compensating charge vacancy formation. The same approach was done previously [10]. The designation OT stands for a Zr atom in an octahedral site (O) and a vacancy localized at the tetrahedral site (T) of the spinel structure. The structure was reoptimized within the GGA PBE level using PAW and a plane waves basis set with a kinetic energy of 435 eV in two ways. First, it was fully optimized, restricted only to FCC. In a second approach, besides keeping the FCC structure, the cell constant was constrained to the values of gamma-alumina. This cell constant was reoptimized in the present work. This last approach is in the spirit of reference 10, in which it was verified that the underlying structure is that of gamma-alumina by X-ray diffraction. So, the ambiance of zirconium is more or less like an impurity in alumina’s subsurface. PAW potentials, used in geometry optimization, are the same used in GW calculations. Brillouin zone summations are carried out using a Monkhorst–Pack (444) *k*-point sampling grid in all calculations, i.e., DFT, GW, and BSE. All calculations were done in VASP. GW calculations were done within the non-self-consistent approximation designated as G_0_W_0_ as implemented in VASP [22]. This choice was not only based on computational convenience but also on the fact it has proved to lead to good estimates of quasi-particle energies. Finally, the spectrum has been simulated by solving the Bethe–Salpeter equation based on GW quasi-energies. The calculation is done in four steps. First, a ground state DFT/PBE calculation. Second, an additional calculation is done to obtain a larger number of virtual bands. The convergence relative to the number of bands should be tested in the final result, since the following step involves a sum over empty states. The third step is the GW calculation and the last is the BSE step.

Convergence with respect to the number of bands was made by increasing the total number of bands for a 4 × 4 × 4 Monkhorst–Pack *k*-point sampling. In all cases, 36 bands are occupied in the ground state. A Lorentzian broadening of 0.1 eV was used in the calculation of the dielectric tensor. Final results were also calculated for 5 × 5 × 5 k-point sampling.

## Results

### Fully optimized structure

The coordinates of the fully optimized structure for the ZrAl_4_O_8_ phase are presented in Table S1 of the supplementary information. The cell constant (8.24 Å) calculated here is slightly larger than the one calculated before (8.15 Å) at LDA approximation [10].

**Fig. 1:**
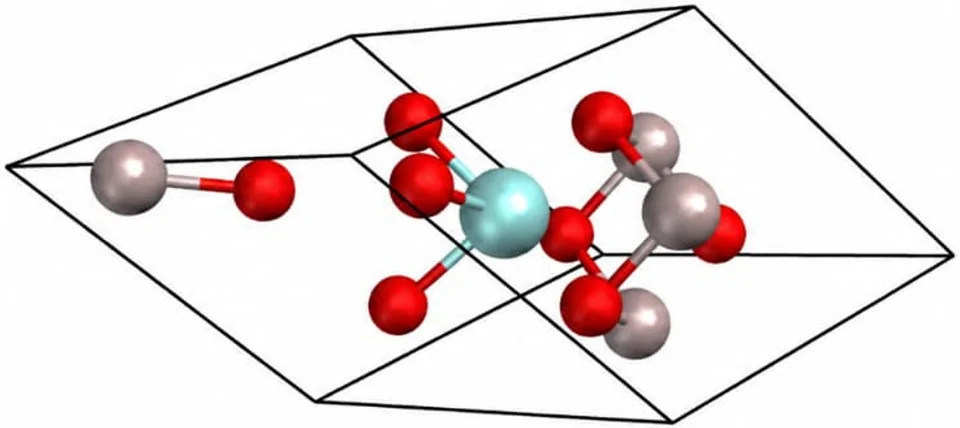
FCC spinel primitive unit cell of the optimized structure of ZrAl_4_O_8_. Atom legend (shown as circles in the image): O, red; Zr, blue; Al, gray. A vacancy lies in the tetrahedral site of the perfect spinel

The structure is shown in Fig. 1. It is worth emphasizing that Zr is in an octahedral site and the vacancy is a missing tetrahedral site in a spinel structure, as explained in detail previously [10].

Out of the optimized structure, GW calculations were performed in order to calculate the bandgap in its non-selfconsistent version, commonly referred to as G_0_W_0_. In Table 1, one can find the gap calculated for three choices for the number of bands in the second step. As mentioned above, the convergence relative to the number of bands should be checked. From 600 to 800 bands, the change in the bandgap is small. The results indicate the value is well converged for 800 bands and, more importantly, our reference value of 5.99 eV agrees with the experimental values of Faro et al. [9], which have found the optical gap in the range of 6.15 to 5.83 eV for samples with increasing zirconium content. Our value is obtained from the difference of quasi-particle energies. Perfect agreement is not expected, since we have calculated the quasi-particle bandgap, while a measurement gives the optical gap.

**Table 1:** Fully optimized structure bandgap calculated for three choices of the number of bands included in the second step of the GW calculation

Number of bands	200	600	800
Bandgap (eV)	5.81	5.96	5.99
Experim. (eV) [9]	5.83–6.15		

The values recorded by Faro et al. [9] are also shown. The range of values refers to samples with different amounts of zirconium relative to the theoretical monolayer

**Fig. 2:**
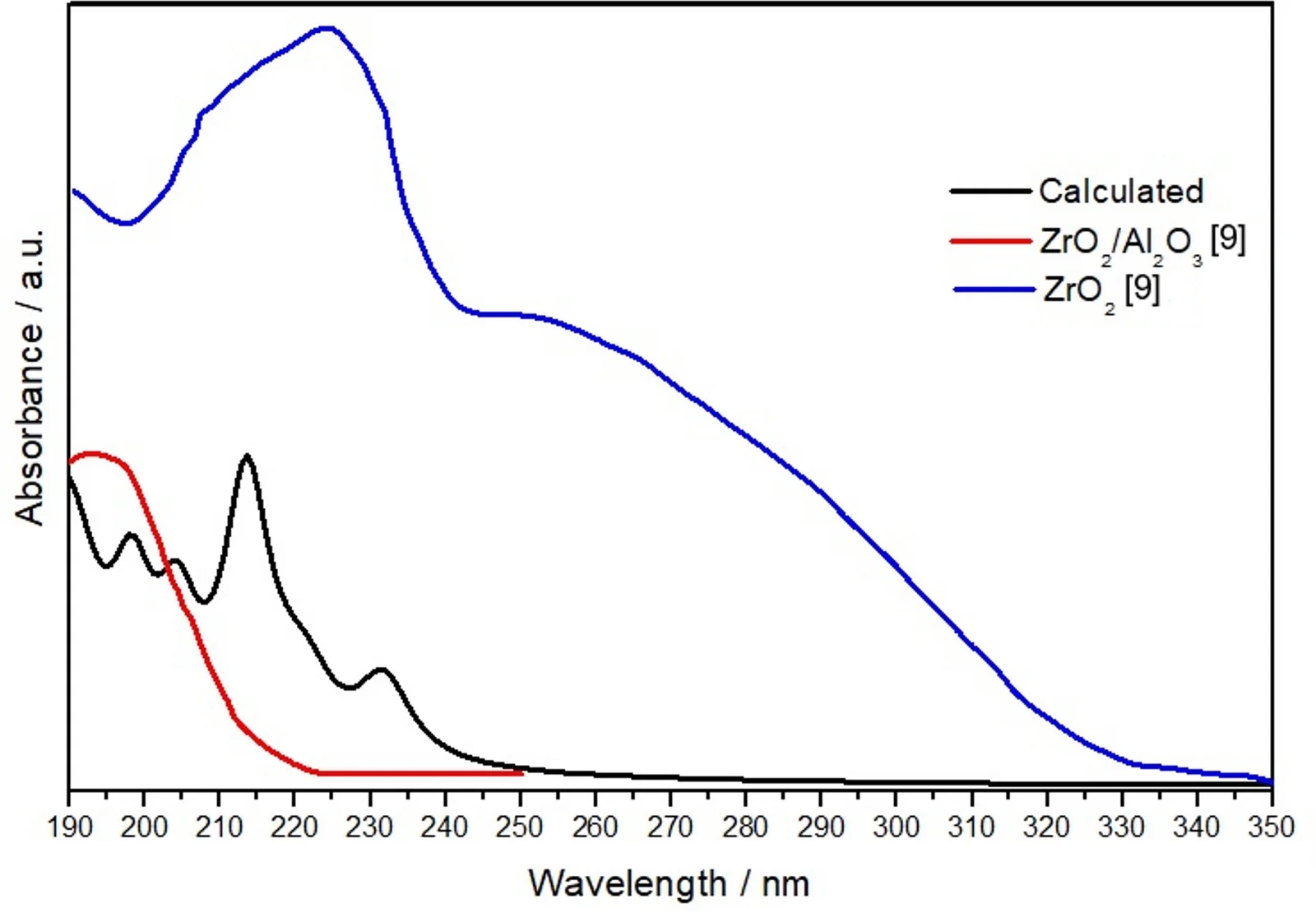
Spectra of UV absorption calculated for the fully optimized structure of ZrAl_4_O_8_ and experimental [9] of ZrO_2_/Al_2_O_3_ and ZrO_2_

Furthermore, we have calculated the spectrum of our model system in the UV region at the GW–BSE level. The result is reported in Fig. 2, compared to the experimental spectrum of Faro et al. [9]. Those authors have pointed out that the spectrum profile of their samples is completely different from that of a sample of pure zirconia, although the XPS results clearly indicate the presence of zirconium in the samples. The results for ZrO_2_ are also shown in Fig. 2 for the sake of completeness. They have also noticed there was excessive noise below ca. 195 nm, due to UV light absorption by atmospheric oxygen. In spite of the fact that the absorption maxima could not be precisely located, due to excessive noise below 195 nm, the authors pointed out it appears to be near this value in all samples.

The calculation with the fully optimized structure does not show good agreement with the experimental spectrum of the ZrO_2_/Al_2_O_3_. Although the absorption is in the same region, there are more defined peaks than the experimental spectrum. It is also clear that it has nothing to do with that of pure ZrO_2_, as should be expected. The experimental spectrum was obtained from diffuse reflectance measurements through the Kubelka–Munk transformation, and the calculation directly accesses the absorption spectrum. Although both approaches are associated with radiation absorption, slight differences in the spectral profile are expected. Nevertheless, the difference in Fig. 2 is more than would be expected. So, we turned to the second approach.

### Structure optimized by constraining the lattice constant to that of gamma-alumina

First, the cell constant of gamma-alumina [21] was optimized to 7.98 Å, which slightly differs from the previous value of 7.95 Å [10]. Then, the cell with formula ZrAl_4_O_8_ was optimized, keeping the constraint of the cell constant at 7.98 Å, i.e., only atomic positions were optimized. The resulting structure can also be generically represented by Fig. 1. The coordinates are shown in Table S2 of supplementary information.

**Fig. 3:**
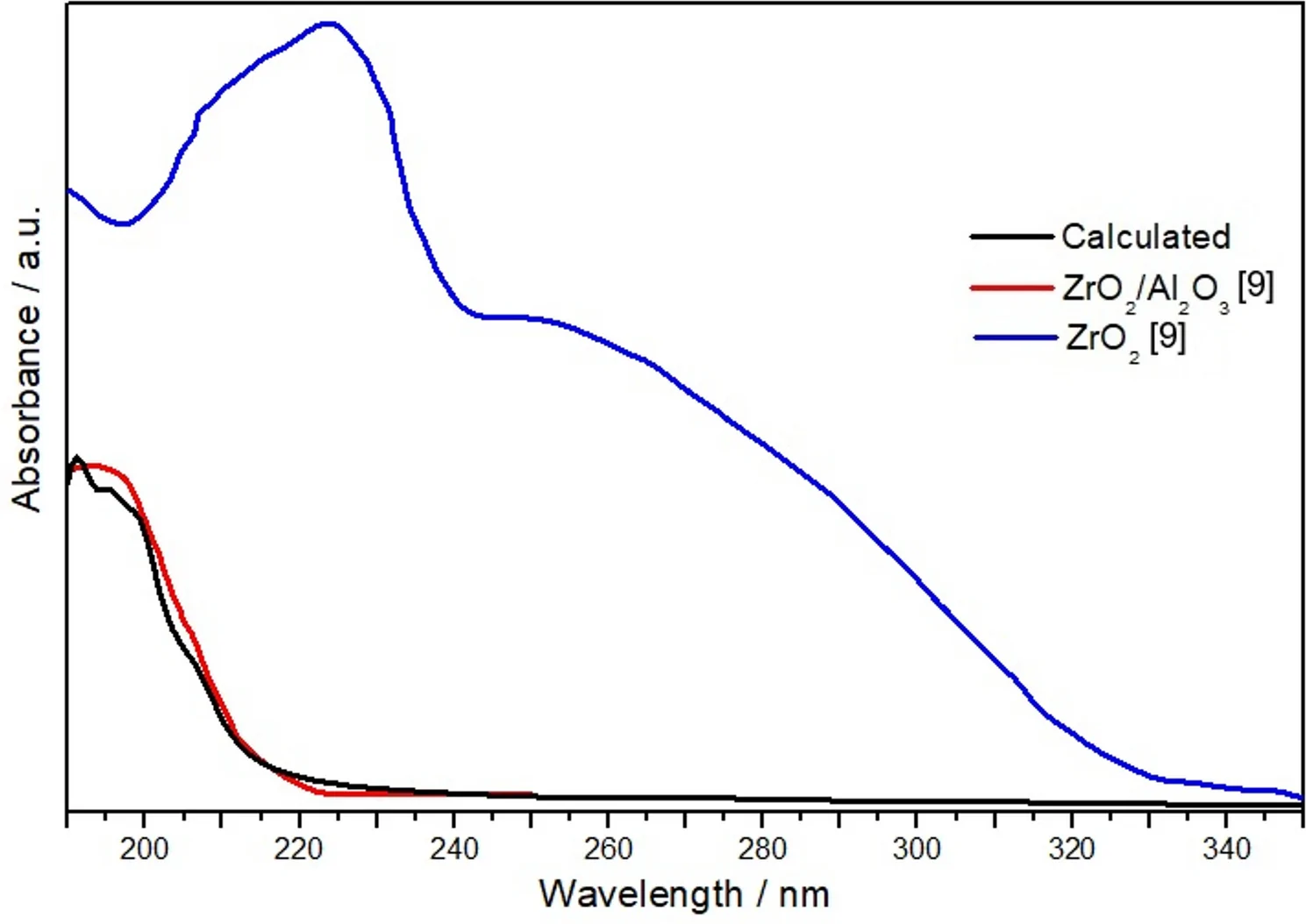
Spectra of UV absorption calculated for the structure optimized constraining the lattice constant to that of gamma-alumina for ZrAl_4_O_8_ and experimental of [9] of ZrO_2_/Al_2_O_3_ and ZrO_2_. A shift of − 10 nm was applied to the calculated spectrum

The calculated absorption spectrum is shown in Fig. 3. As can be seen, after a shift of − 10 nm, the agreement with the experimental profile of ZrO_2_/Al_2_O_3_ is very good. This shift is, in the relevant part of the spectrum (190–200 nm), about 0.3 eV in the energy scale, which means that the error is small, comparable to the best ab initio methods in quantum chemistry.

On the other hand, the gap obtained by quasi-particle energy is slightly larger in this last case, i.e., 6.47 eV. As already mentioned, a perfect agreement is not expected. The convergence of the gap with the number of bands is shown in Table 2.

**Table 2:** Constraint optimized structure bandgap calculated for three choices of the number of bands included in the second step of the GW calculation

Number of bands	200	600	800
Bandgap (eV)	6.23	6.43	6.47
Experim. (eV) [9]	5.83–6.15		

The values recorded by Faro et al. [9] are also shown. The range of values refers to samples with different amounts of zirconium relative to the theoretical monolayer

In general, our calculations show good agreement with the spectrum profile reported by Faro et al. [9]. The spectrum profile agrees quite well with the experiment for the cell with the lattice constraint by the gamma-alumina value. The position is shifted by 10 nm. This discrepancy can be attributed to at least two possible reasons. Of course, it can be attributed to the limitations of the method, such as the use of PBE in the DFT part of the calculation. But an additional point concerning the reported experimental values should also be considered. The experimental spectrum was obtained from diffuse reflectance measurements through the Kubelka–Munk transformation. Although both approaches are associated with radiation absorption, slight differences in the spectral profile are expected, as already mentioned.

Some qualitative aspects can be viewed in the projected density of states and the DFT band structure as shown in Figs. S1 and S2 of the SI. The first indicates that the Zr-3d orbitals contribute to the conduction band, but not to the valence band. This latter is mainly composed of oxygen p bands. The second indicates a direct gap at the gamma point.

**Fig. 4:**
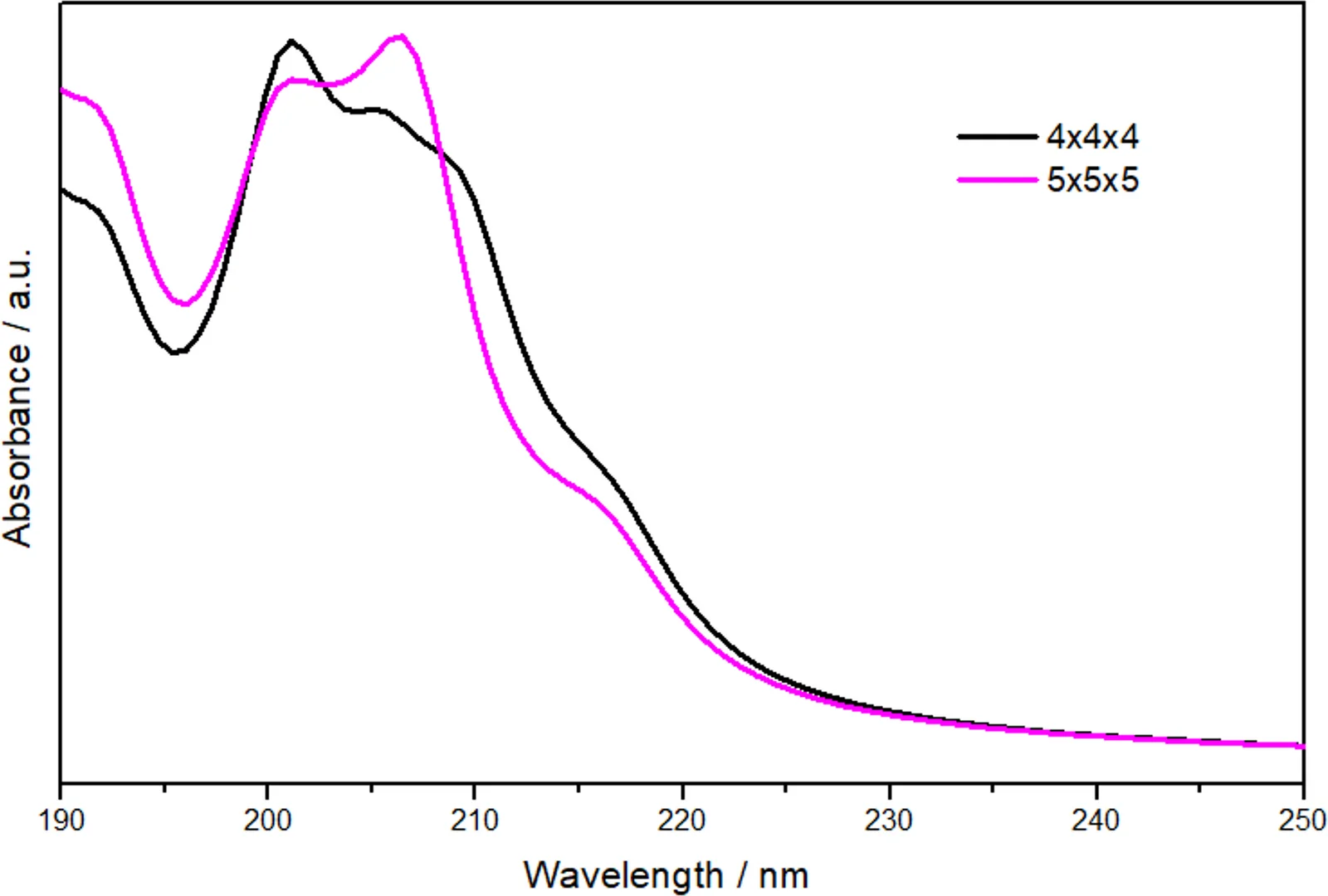
Spectra of UV absorption calculated for the structure optimized constraining the lattice constant to that of gamma-alumina for ZrAl_4_O_8_ for two *k*-point samplings. No shift was applied

Finally, the dependence of the spectrum profile was also verified for 5 × 5 × 5 k-point sampling. The comparison with 4 × 4 × 4 is shown in Fig. 4. The general profile of the results is similar, although the relative intensities of some peaks change, showing a residual dependence on *k*-point sampling, which does not change the general trend though.

## Conclusions

The present study provided a theoretical investigation of the optical properties of the zirconia–alumina mixed oxide, specifically ZrAl_4_O_8_, through DFT combined with GW–BSE calculations. The optimized structure enabled the simulation of the UV absorption spectrum with satisfactory agreement with experimental data reported in the literature, confirming the reliability of the computational methodology employed for describing the optical behavior of the solid.

The absorption is in the ultraviolet region. Furthermore, the inclusion of electron–hole interactions through the Bethe–Salpeter formalism proved essential for accurately reproducing the spectral features and optical transitions of the material.

The structure obtained by constraining the lattice constant to be that of gamma-alumina better represents the ZrO_2_/Al_2_O_3_ phase, which, instead of representing a dispersion of zirconia in alumina, is the result of the migration of Zr^+4^ to the subsurface of alumina. That is the same conclusion as ref. 10. In the present work, as well as in ref. 10, the phase is approximately represented by a defective spinel cell with the formula ZrAl_4_O_8_.

These findings contribute to a deeper understanding of the relationship between the structural organization and the optical response of zirconia–alumina mixed oxides, highlighting the influence of mixed oxide formation on the electronic structure of the system. In addition, the good agreement between theoretical and experimental results demonstrates the potential of advanced first principles approaches as predictive and rationalization tools for the investigation of similar materials.

## Supplementary Information

Below is the link to the electronic supplementary material.

ESM 1 (PDF 243 KB)

## Data Availability

No datasets were generated or analysed during the current study.
