# Supplementary material for: Accurate prediction of UV absorption in zirconia–alumina mixed oxide (ZrAl4O8) via GW–BSE calculations
Source: J Mol Model. 2026 Sep 9;32(10):355. doi: 10.1007/s00894-026-06939-6 (PMC13558391; doi:10.1007/s00894-026-06939-6)
Supplement: Supplementary file 1 [file 894_2026_6939_MOESM1_ESM.pdf]

Supplementary Information

Accurate Prediction of UV Absorption in Zirconia–Alumina Mixed Oxide ($ZrAl_4O_8$) via GW–BSE Calculations

Table S1: Reduced coordinates of optimized FCC structure of the $ZrAl_4O_8$ (a = 8.24 Å).

Atom	Coordinates		
Zr	0.493	0.493	0.493
Al	0.879	0.879	0.879
Al	0.016	0.492	0.492
Al	0.492	0.016	0.492
Al	0.492	0.492	0.016
O	0.265	0.265	0.265
O	0.713	0.252	0.252
O	0.252	0.713	0.252
O	0.252	0.252	0.713
O	0.744	0.744	0.744
O	0.267	0.756	0.756
O	0.756	0.267	0.756
O	0.756	0.756	0.267

Table S2: Reduced coordinates of optimized FCC structure of the $ZrAl_4O_8$ constraint to gamma-alumina lattice constant (a = 7.98 Å).

Atom	Coordinates		
Zr	0.492	0.492	0.492
Al	0.877	0.877	0.877
Al	0.013	0.493	0.493
Al	0.493	0.013	0.493
Al	0.493	0.493	0.013
O	0.265	0.265	0.265
O	0.721	0.248	0.248
O	0.248	0.721	0.248
O	0.248	0.248	0.721
O	0.744	0.744	0.744
O	0.267	0.756	0.756
O	0.756	0.267	0.756
O	0.756	0.756	0.267

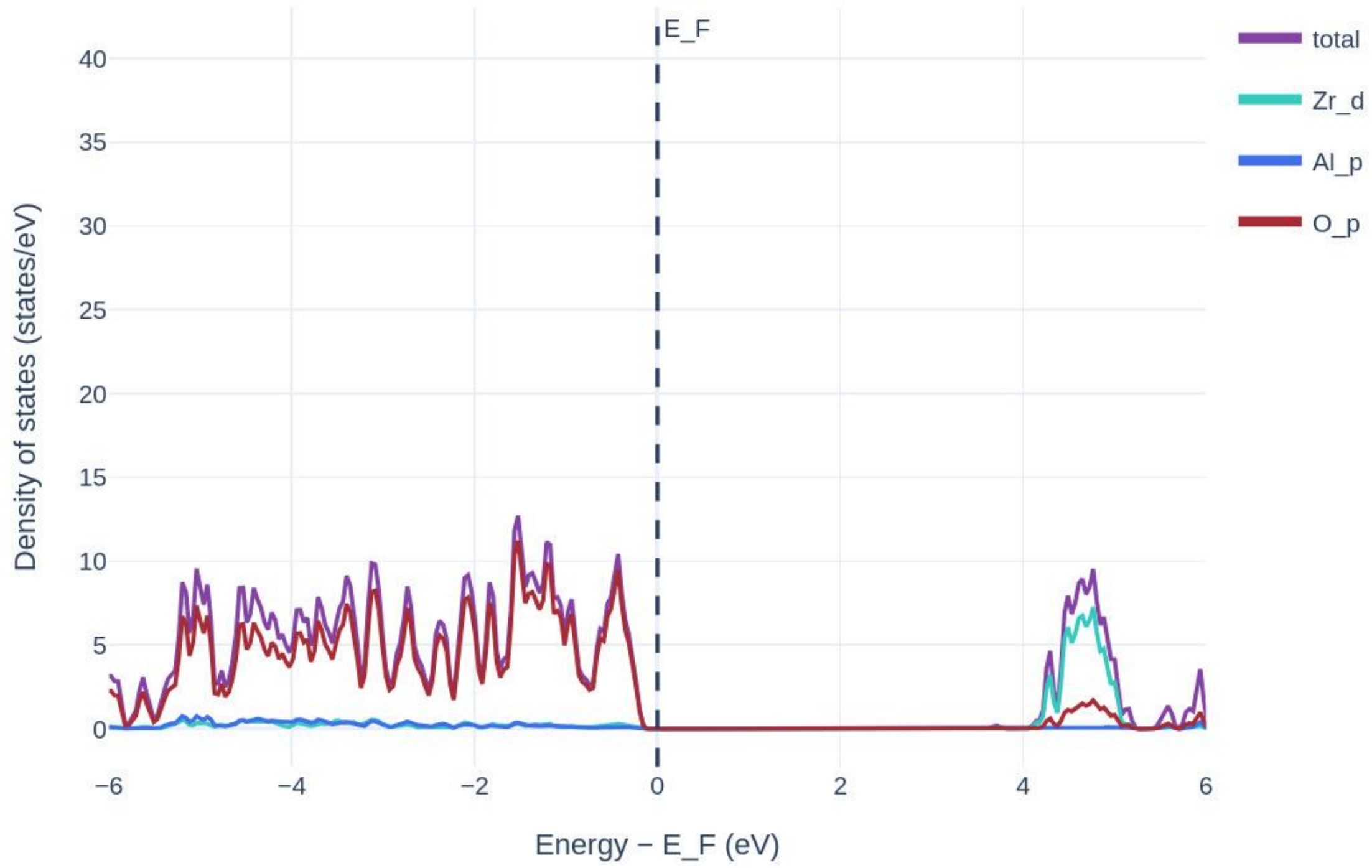

Fig. S1: Projected density of states for $ZrAl_4O_8$ in constrained optimization.

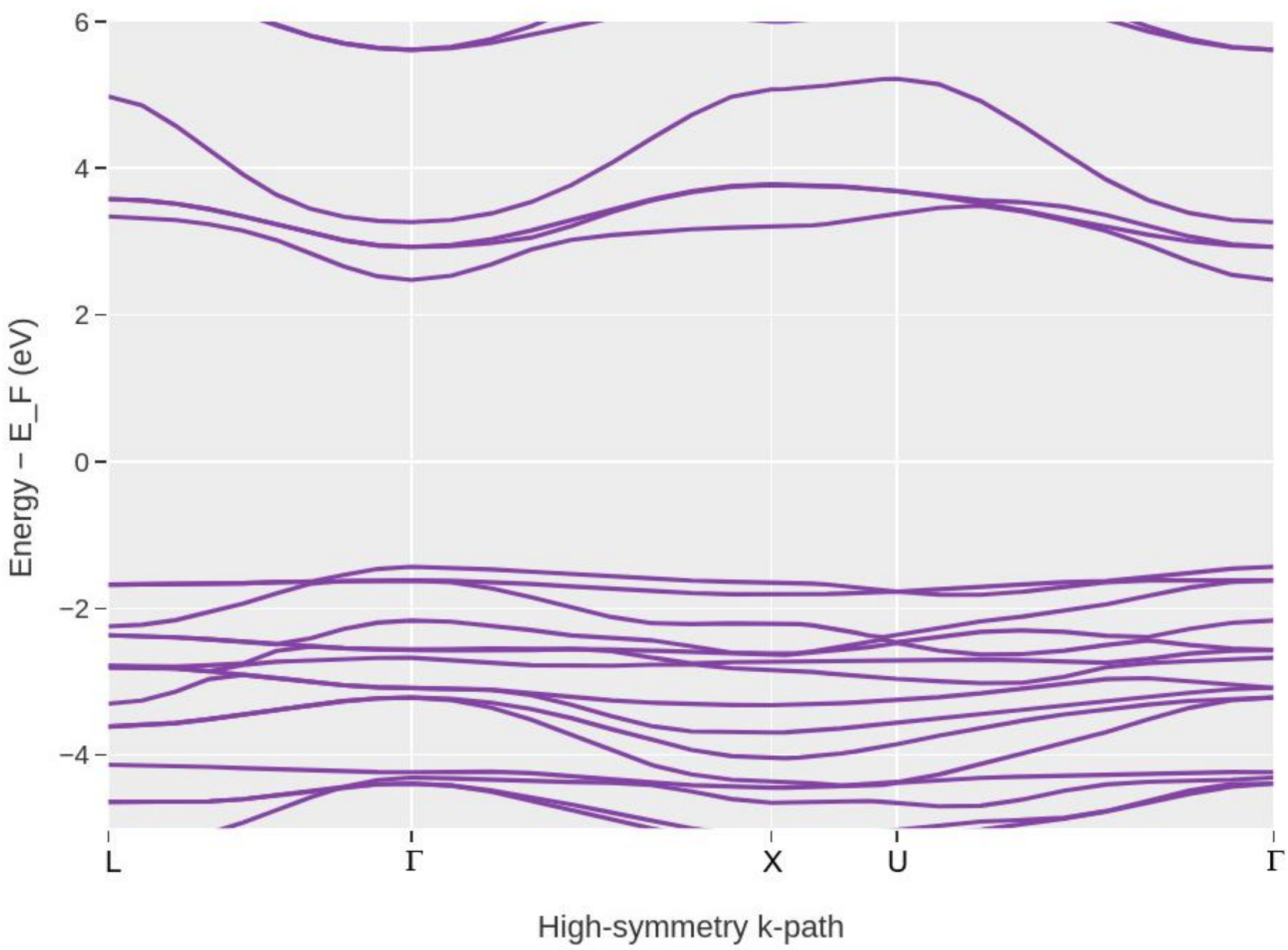

Fig. S2: Band structure for $ZrAl_4O_8$ in constrained optimization.
